# State Board of Health

**Published:** 1875-09

**Authors:** Joseph P. Logan

**Affiliations:** Chairman of Prudential Committee


The State Board of Health has recently issued, through a committee, an address to the medical profession of the State, setting forth in a clear and forcible light the objects of the organization and the ends to be attained by its labors. The organization of this Board is the consummation of much labor upon the part of prominent and earnest members of the profession in Georgia who have led the sentiment of the masses upon the subject of State registration and public hygiene for many long years. The whole profession is deeply interested in the success of this movement; and as the usefulness of this Board must depend upon the manner in which it is supported by the great mass of the profession in the State, we trust that all will join heartily in support of the movement and gladly discharge each his own duty in the premises, as well as extend to the Board every courtesy, sympathy and encouragement in their public-spirited and gratuitous labor. We copy from the pamphlet above mentioned the following:

SPECIAL REQUEST.

The chairmen of the following standing committees earnestly request the favor of prompt assistance from their professional brethren, in furnishing *data, however brief*, for the preparation of their several reports, to be made in October next:

Endemic, Epidemic and Contagious Diseases—Report to Dr.

Campbell their extent of region, their character, number of cases, fatality and season of greatest prevalence.

Hygiene of Schools, Prisons and Public Institutions—Report to Dr. Nottingham the sanitary condition of such institutions, with the kinds and amount of diseases, and degree of mortality; noticing also any points of excellence, or any obnoxious features incident to their management.

Geology, Topography, &c.—Report to Dr. Little all peculiarities of geology and topography which may have a bearing on the health of the locality in which you reside.

Poisons and Special Sources of danger—Report to Dr. Stanford all facts upon the subjects confided to this committee.

Report to Dr. Logan, chairman of Special Committee, all facts bearing on vaccination and means of suppressing small pox.

JOSEPH P. LOGAN,
*Chairman of Prudential Committee.*

